# Perspectives on research needs in healthcare epidemiology, infection prevention, and antimicrobial stewardship: what’s on the horizon—Part II

**DOI:** 10.1017/ash.2023.474

**Published:** 2023-11-16

**Authors:** Jonas Marschall, Rachael E. Snyders, Hugo Sax, Jason G. Newland, Thais Guimarães, Jennie H. Kwon

**Affiliations:** 1 Division of Infectious Diseases, Washington University School of Medicine, St. Louis, MO, USA; 2 BJC Healthcare, St. Louis, MO, USA; 3 Bern University Hospital, University of Bern, Bern, Switzerland; 4 Division of Infectious Diseases, Department of Pediatrics, Washington University School of Medicine, St. Louis, MO, USA; 5 Infection Control Department, Hospital das Clínicas, University of São Paulo, São Paulo, Brazil

## Abstract

In this overview, we articulate research needs and opportunities in the field of infection prevention that have been identified from insights gained during operative infection prevention work, our own research in healthcare epidemiology, and from reviewing the literature. The 10 areas of research need are: 1) Transmissions and interruptions, 2) personal protective equipment and other safety issues in occupational health, 3) climate change and other crises, 4) device, diagnostic, and antimicrobial stewardship, 5) implementation and deimplementation, 6) healthcare outside the acute care hospital, 7) low- and middle-income countries, 8) networking with the “neighbors,” 9) novel research methodologies, and 10) the future state of surveillance. An introduction and chapters 1–5 are presented in part I of the article and chapters 6-10 and the discussion in part II. There are many barriers to advancing the field, such as finding and motivating the future IP workforce including professionals interested in conducting research, a constant confrontation with challenges and crises, the difficulty of performing studies in a complex environment, the relative lack of adequate incentives and funding streams, and how to disseminate and validate the often very local quality improvement projects. Addressing research gaps now (i.e., in the post-pandemic phase) will make healthcare systems more resilient when facing future crises.

We identified 10 areas of research need (Table 1); part I of the review covers topics 1–5, and part II covers topics 6–10.

**Table 1.** Research needs and opportunities in infection prevention and control (IPC)

Chapter	Title	Addressed topics
1	Transmissions and interruptions	Transmission concepts, interventions to interrupt the transmission chain, environmental cleaning, decolonization
2	PPE and other safety issues of occupational health	Protective equipment, vaccination, hand hygiene
3	Climate change and other crises	Effect of climate on SSI, antimicrobial resistance (AMR), zoonoses, emerging infections, human encroachment into remaining biotopes, how to strengthen healthcare for crises, sustainability
4	Device, diagnostic and antimicrobial stewardship	Stewardship forms, device reprocessing
5	Implementation and deimplementation	Behavioral change, learning and unlearning, discontinuing low evidence measures
6	Healthcare outside the acute care hospital	Outpatient care, nursing homes, long-term care facilities
7	Low- and middle-income countries	Inequality and scarcity, low-cost interventions, knowledge transfer, travel screening
8	Networking with the neighbors	Collaborations with related fields, such as patient safety and quality, microbiology and data science; explore the interfaces between areas
9	Novel research methodologies	Emulated trials, cluster-randomized trials (CRT), machine learning, design research networks, artificial intelligence
10	The future state of surveillance	Early detection, automated surveillance, optimizing the feedback loop

Note. The research needs are presented in topical groups with overarching labels; notably, there is overlap between many of these groups.

## Chapter 6—Healthcare outside the acute care hospital

One of the remaining frontiers in infection prevention and control (IPC) in high-income countries is the outpatient world. As care shifts from inpatient to ambulatory medicine (and day surgery), so do complications. We currently have inadequate evidence on how to prevent outpatient healthcare-associated infections (HAI) that have its root in a usually brief healthcare encounter. This includes central line-associated bloodstream infection in outpatient parenteral antibiotic therapy patients, catheter-associated urinary tract infection in those with long-term catheterization, surgical site infection (SSI) in outpatient surgery, but may also involve other populations such as patients receiving joint injections for osteoarthritis pain management. Of note, it is unclear how to best capture these outpatient HAIs, as traditional surveillance systems would require a significant effort.^1,2^ Possibly, modern surveillance by means of patient-operated smartphone applications, ICD-10 codes of follow-up encounters or claims data, could help in identifying HAI events in these outpatient populations.

Sectors that require more dedicated research are long-term care facilities (LTCF) and nursing homes (NH). Neglected by IPC activities due to lack of personnel and financial resources (e.g., fewer cultures taken because of the costly microbiology work-up), there are many improvements to be made in these settings. The link from LTCF/NH to acute care hospitals is particularly intriguing as there is a constant transfer of patients in both directions; if one side does not identify the MDRO-colonized patient, they may inadvertently trigger an outbreak on the other side. How to reach and train the LTCF/NH workforce in infection prevention and antimicrobial stewardship is a major challenge.^3^

## Chapter 7—Low- and middle-income countries

Information that stems from studies conducted in high-income countries may not necessarily translate to LMIC settings, given that the epidemiology often differs and both infrastructure and resources can be limited. Accordingly, IPC strategies have not been tailored to LMIC as much, and its benefits have not reached all areas of the world equally.^4^ This is problematic and should lead to a push to bring cutting-edge research to LMICs. Although there are funding opportunities dedicated to resource-limited settings, such as those issued by the NIH Fogarty International Center, IPC research remains heavily underfunded in comparison to other infectious diseases research. Not all research would necessarily be costly, nor are all preventive measures expensive. Some elements rely on the local work culture, which may be amenable to low-cost interventions in terms of addressing HCP behavior (but with the potential for high yield). The same goes for areas of conflict: a recent qualitative study on how to best implement measures cited low-cost interventions such as having IPC champions and offering illustrated guidelines to healthcare workers.^5^ Knowledge sharing and knowledge sharing networks are key in disseminating information and should be cultivated in LMIC, if they do not already exist.^6^ Regarding COVID-19, there is a good overview of research deemed necessary to reduce its influence on LMIC.^7^

LMIC are often the first victims of outbreaks as humans encroach on remaining pockets of untouched nature. A recent example of an illness spreading from an endemic area in an LMIC setting to other continents is the 2022 mpox outbreak. In the past, mpox has usually been presented as an endemic curiosity in tropical medicine textbooks. Confined to a small geographic area until recently (with very few exceptions), it stands for the neglected diseases of LMIC settings that have the potential for widespread outbreaks. The implicit theme of “equality in access to the best possible care while ensuring patient safety” may be beyond the influence of IPC experts. A noteworthy publication in the wake of the COVID-19 pandemic suggested striving for equal access to care, soliciting greater solidarity among the nations and working toward universal preparedness for challenges.^8^

More research is needed on the contribution of anthropological and socioeconomic factors to the global antimicrobial resistance and how to counter this threat best. Collignon et al used bacterial resistance and antimicrobial consumption databases and correlated the data with World Bank indicators such as governance, education, gross domestic product per capita, healthcare spending, and community infrastructure (e.g., sanitation). They conclude that improving sanitation, increasing access to clean water, and ensuring good governance, as well as increasing public healthcare expenditure and better regulating the private health sector are all necessary to reduce global antimicrobial resistance.^9^

Lastly, in terms of global travel to and from LMIC, the optimal form of screening patients following travel is unclear and should be characterized better. The risk of carrying multidrug-resistant organisms is influenced by factors such as area of travel, level of exposure to the local healthcare system, and antibiotic receipt.

## Chapter 8—Networking with the “neighbors”

Innovation is known to occur more readily at the interface of different areas, and specifically, when different thoughts collide. It is therefore important to be aware of the neighboring fields and identify research questions that can be answered by the collaboration between IPC and the respective partner.

An example from our own experience is a study into the effect of operation room ventilation on SSIs, for which we worked with a team of ventilation engineers.^10^ Owing to this collaboration, we were able to identify the need for a straightforward descriptor of ventilation quality in operating rooms. Very likely, this need would not have been recognized by either collaboration partner alone. As such, we would like to reframe IPC research as one set to benefit immensely from interdisciplinary work. Stimuli may come from (but are not limited to) statistics, data science, engineering, environmental sciences, psychology, economics, behavioral science, human factors, disinfection and sterilization, quality management, patient safety, anesthesiology, microbiology, information technology, and a wide array of specialty areas such as, for example, nutritional science (e.g., the effect of malnutrition on SSI risk^11^ and how to correct this). Each of these areas is evolving, and new developments can trigger research ideas that were not (or could not be) addressed before. For example, the broader availability of whole genome sequencing in partnership with microbiology laboratories has made outbreak investigations much more granular and now permit tracing entire evolutionary pathways.

Although research in IPC has traditionally come from research-heavy academic medical centers, there is a need to install more inclusive, larger research networks. These should include rural sites. Although large surveillance systems can provide representative data on a condition, including pre/post data in intervention studies, they are usually hampered by the limited number of available variables. We believe that more research networks should be formed to address questions with the appropriate statistical power (as for example, the CDC Prevention Epicenters program does, https://www.cdc.gov/hai/epicenters/index.html).

This discourse touches on how to position IPC as an *expert group for complex systems*, which is what contemporary healthcare is. Very few other professionals are so well connected with other players inside a healthcare organization. This also predisposes IPC teams to conduct research on safe processes that are the product of multidisciplinary work (for example, IPC might evaluate the microbial contamination of stem cell products, what consequences this has on a patient receiving transfusion, and if antibiotic prophylaxis is required). In that role, IPC should encourage and help everybody across a healthcare system who does QI work related to infection prevention to disseminate their results so that others outside that institution can benefit from novel insights.

From the perspective of funding mechanisms, IPC research could theoretically be funded by nearly every National Institutes of Health (NIH) center (and others entities such as, for example, the Agency for Healthcare Research and Quality, AHRQ, and the Patient-Centered Outcomes Research Institute, PCORI), as it affects all aspects of modern medicine. This seems attractive at first sight but makes navigating the grant landscape more difficult than if there was a straightforward match (e.g., the NIDDK institute for nephrology research). We hope that funding agencies are cognizant of the fact that there is no dedicated center for infection prevention (yet) and therefore no natural “home” for IPC research.

## Chapter 9—Novel research methodologies

Not all research can be pursued by conducting randomized controlled trials; we anticipate that much evidence will come from time-series analyses, before/after studies, and other designs popularized in epidemiology research in recent years such as stepped wedge studies.^12,13^ Cluster-randomized cross-over trials are an elegant way around randomization of individual patients, which often is neither feasible nor sensible in IPC. In a recent example of such a trial groups of patients in five hospitals were given either 24 or 24–48 h of postoperative antibiotics, with group assignments switching every 2–4 mo (and prolonged antibiotics did not confer additional risk reduction in developing SSI).^14^ More studies should employ this design, and it is particularly well suited for multicenter trials.

Often, however, it will be difficult to set up trials, for one because they are costly and take extended times for planning and conducting them. Second, because the outcomes may be so infrequent that a trial becomes unattractive to even begin. Target trial emulation is a novel approach that relies on available observational data and then models a comparative study. Recent examples are on vaccine effectiveness against COVID-19.^15,16^

There are many other trial approaches that deserve to be inspected by IPC researchers, including designs such the “personalized randomized controlled trial”^17^ or the “durations design”.^18^ Another innovative study design is the Sequential Multiple Assignment Randomized Trial (SMART) that is suitable for IPC work where usually multiple contextual factors are at play and a bundle of preventive measures may be needed to achieve the desired outcome.^19^

Qualitative research, with its roots in sociology and psychology, has the potential to elicit mental models of providers and patients and help us understand what drives our behavior. Video-reflexive ethnography is a special form of qualitative research that involves filming healthcare practices and examining the material to detect weak points. Human factors engineering is about conceiving an environment, tools, and processes around human beings to improve our “output.”

Lastly, data science and the first steps in artificial intelligence are visible in hospital epidemiology. A recent example involves outbreak investigation.^20^ Beyond the local setting, applications in global scenarios are within reach.^21^ As more routine medical data become available in dedicated data science centers in healthcare, innovative approaches for using and visualizing it should be employed. These data should be supplemented with sensor-generated information (such as on whether a handrub dispenser was used or not).

An extreme form of putting data to use is the mathematical modeling of pathogen transmission and HAI acquisition.^22^ These stochastic models are usually grounded in real-world data and rely on multiple assumptions to produce output. They may help with estimating the impact of public health interventions, trial planning, projections of disease burden, and more.

## Chapter 10—The future state of surveillance

Surveillance activities take time and effort from the IPC team, including manual chart review, data cleaning, reporting back to the surveilled units, and communication with public health authorities. Future research should inspect ways to make this process more efficient. Automated (or semi-automated) surveillance of HAI is a result of the move to electronic medical records (EMR). The potential time freed up for IPC personnel by transitioning to electronic surveillance is considerable, with an estimated 74% reduced workload.^23^ However, dedicated time from hospital informatics is quintessential and not always offered, algorithms for different EMR platforms have to be developed, and variables relevant for surveillance need to be identified. Once surveillance becomes less time consuming, IPC personnel can dedicate more time to improvement projects. A variation of this is to identify surrogate markers for HAI; for example, ICD-10 codes for postoperative infections could be utilized to determine where detailed surveillance is needed. Yet, approaches based on nonclinical data sources need to be critically reviewed before implementation.^24^

Another aspect of surveillance that deserves more attention is the early detection of outbreaks. This can be in the form of microbial species not otherwise seen (such as emerging infections), common species becoming more frequent, or certain resistance determinants increasing. Rapid diagnostic methods may help with surveillance, but platforms are heterogeneous and availability can be an issue.^25^ Also, HAIs that are not subject to mandatory surveillance and public reporting may increase in frequency and go undetected for some time. Our tools for early detection are limited, and we are likely to notice only what we have decided to measure. Algorithms to detect upticks of infections need to be developed and promise to facilitate our work.^26^ Again, pattern recognition tools could incorporate EMR variables such as billing codes, microbiology lab trends, and free text crawling for terms suggestive of outbreaks, and they should explore artificial intelligence.

On an international scale, there are tools that allow early reporting (such as promedmail.org); however, it is difficult to identify the most relevant threats early on, given the noise of information. HAI and AMR data need to be synthesized across regions and countries so they can serve for benchmarking and as outcomes for large public health intervention studies.

Furthermore, surveillance should not be a purpose to itself (and surveillance definitions should not be too different from what is considered a clinical diagnosis of infection) but provide data that is fed back to the providers in a digestible way and then leads to improvements; as such, the mechanics of the feedback loop should be a topic of study. Surveillance should always be actionable.

## Discussion

The 10 overarching themes of IPC research needs presented here reflect current megatrends: climate action and sustainability, digitalization, inequality, demography, urbanization, health and nutrition, and lastly, migration.^27^ Specific for ID, overarching trends are emerging and re-emerging diseases, antimicrobial resistance, demographic changes, and technological advances.^28^ Both the review of current evidence and the field of “futures study” can help us identify gaps in knowledge and highlight research opportunities.^29^

There are considerable barriers in developing IPC further, along with its research agenda. One is that new insights depend so much on the local culture in how fast they can be absorbed into clinical reality and serve for the institution to provide better patient outcomes; the local culture may not be ready for this change. Another barrier is that much of the innovative research is in fact day-to-day quality improvement work that never gets disseminated outside of a given institution, which may be due to lack of training, time, and suitable incentives. How to learn from all healthcare systems, even the smallest ones, is therefore a goal that we should pursue. Third, we IPC professionals are constantly putting out fires and should at the same time make our healthcare institutions safer. The parade of challenges and crises can in itself strengthen a system, but they often prevent us from proactive conceptualization and developing proposals on much needed research. Fourth, the lack of maturity of the IT environment can prevent meaningful research from being carried out, due to the heterogeneity of EMR platforms, fragmented IT solutions, and the frequent lack of informatics support.

In addition, how to train, motivate, and retain the IPC workforce to contribute to the advancement of knowledge is of tremendous importance. Yet, there are more financially appealing careers for nurses than to go into IPC, and the drought of young doctors opting for a career in ID has become evident in 2022 when only 56% programs in the U.S. filled all their fellowship slots. In addition, the largely *cognitive specialty* of IPC is providing additional value to an institution that often goes unnoticed and uncompensated. We need to invest more in the next generation and attract talent so that innovative research can go on. One key aspect in this is the display of “avoided infections” and their pecuniary after-effect, i.e., the “cost avoided,” as a product of IPC work.

This review has one major limitation in that it is the subjective work of a group of IPC experts, and not a systematic review or meta-analysis. However, an overview of research needs in IPC is a glimpse into a possible future of the field and as such exploratory in character. These are exciting times to contribute to IPC research as the field has been tested by a pandemic and now will enter a new phase and, possibly, growth. Our profession’s goal of making healthcare safer from an infection prevention standpoint is more relevant than ever; addressing research gaps now (i.e., in the post-pandemic phase) will make healthcare systems more resilient when facing future crises.
